# The Burden of Living With Cutaneous Lupus Erythematosus

**DOI:** 10.3389/fmed.2022.897987

**Published:** 2022-08-08

**Authors:** Cristina Drenkard, Kamil E. Barbour, Kurt J. Greenlund, S. Sam Lim

**Affiliations:** ^1^Division of Rheumatology, Department of Medicine, Emory School of Medicine, Atlanta, GA, United States; ^2^Department of Epidemiology, Rollins School of Public Health, Emory University, Atlanta, GA, United States; ^3^Centers for Disease Control and Prevention, Atlanta, GA, United States

**Keywords:** quality of life, psychosocial impact, racial minorities, cutaneous lupus erythematosus (CLE), disease burden

## Abstract

Cutaneous lupus erythematosus (CLE) is a group of heterogeneous autoimmune disorders primarily affecting the skin. Patients with these conditions are mostly young women when they become sick and often suffer from recurrent skin symptoms or longstanding changes in their physical appearance. CLE disorders lead to different levels of morbidity and can impact profoundly patients' quality of life, particularly in the psychological and social health domains. This review provides a summary of recent research investigating the psychosocial burden of living with CLE and the intersect amongst the disease characteristics, patient factors, and social determinants of health. Furthermore, this review provides insight into patient care and research needs that remain unmet to improve the quality of life of patients living with CLE.

## Introduction

Cutaneous lupus erythematosus (CLE) is a group of heterogeneous autoimmune disorders primarily affecting the skin and mucosal tissue, showing varying levels of association with systemic lupus erythematosus (SLE). CLE comprises multiple conditions classified into three major subgroups based on the disease morphological characteristics and chronicity: acute (ACLE), subacute (SCLE), and chronic (CCLE) cutaneous lupus erythematosus (1, 2). ACLE consists of transitory erythematosus rashes, which are often localized on the malar area of the face, also known as “butterfly” rash, on UV-exposed areas, or as a generalized rash. SCLE typically presents as an annular or a papulosquamous rash on photo-exposed areas of the trunk and arms. SCLE rashes last longer than ACLE and can cause dyspigmentation. CCLE is the largest subgroup and includes multiple distinctive conditions, including discoid lupus erythematosus (DLE), lupus panniculitis, chilblain lupus, and lupus tumidus. CCLE subtypes can cause scaring and are less likely to be associated with SLE than ACLE and SCLE. DLE, the most common subtype, is characterized by erythematous discoid-shaped, adherent plaques and papules that can be localized in any area of the body, but are more likely to be on the scalp, ears, and face. DLE heals causing dyspigmentation, atrophy, scarring, and permanent hair loss (1, 3, 4).

CLE affects all age groups but is rare in children, and is more common in females with different proportions according to subtype. The female to male incidence ratio ranges between 3:1 and 4:1 for CLE as a group, and between 3:1 and 8:1 for DLE (5–8). Population-based studies indicate that Black people develop the disease at younger age than White people. The mean age at DLE diagnosis was 48.5 and 53 years-old in the predominantly White populations of Olmstead County, Minnesota and Sweden (6, 9), respectively, and 32 years-old in the African-descendent population of French Guiana (10).

There are also racial disparities in the incidence, morphology, and severity of CLE subtypes. While SCLE is more likely to occur in White individuals (11), CCLE, in general, and DLE, in particular, disproportionately affect Black individuals. In the Southeast USA, where the population is evenly distributed between White and Black people, the overall incidence of CCLE and DLE was reported to be at a minimum of 3.9/100,000 and 3.7/100,000 person years, respectively (8). CCLE and DLE incident rates were 3.9- and 4.1-fold higher for Black compared to White people, respectively. Racial disparities were also reported in the prevalence of DLE in Manhattan, with higher rate of cases per 100,000 persons-year among Blacks (23.5) and Latinos (8.2) compared with Whites (1.8) and Asians (0.6). The average age at diagnosis was lowest among Black people (36.7 years old) and highest among White people (63.4 years old), whereas Latino and Asian people were in average 45.8 and 45.3 years old, respectively (7). Among CLE patients at the University of Pennsylvania, Black people had more skin damage at onset and during follow-up than White patients (12), while Black patients with DLE from Texas had significantly worse damage at baseline and greater risk of dyspigmentation at any anatomical location than those of other race/ethnicity (13).

## The Burden of CLE

### Patient Perspectives of Living With CLE

Two recent qualitative studies have shed light on how CLE may be perceived by patients and how the disease may affect patients' lives (14, 15). The most salient themes include the negative impact of living with CLE on patients' mental health, which can lead to social anxiety, maladaptive responses, and negative coping strategies such as recreational drug use (14). Issues related to physical signs and symptoms, including scarring and dyspigmentation, fear of disease progression, body image and self-consciousness are often elicited by patients (15). Qualitative findings suggest that the emotional distress caused by living with CLE persists in a large majority of patients, regardless of the disease duration; however; patients' concerns may differ by demographic characteristics (14, 15). White patients reported predominantly fear of disease progression and physical signs and symptoms, whereas Black patients often elicited self-consciousness, alopecia and dyspigmentation. Furthermore, patients aged 60 or younger were more likely to report emotional symptoms than older patients (15).

Individuals living with CLE report that their personal relationships are profoundly affected (14). Patient testimonies indicate high levels of distress about their appearance as well as being socially stigmatized (15). Self-consciousness, one of the most common themes among CLE patients, is intensified by comments made to the patient by other people. These conditions also interfere with outdoor activities due to photosensitivity. Patients often report feelings of helplessness and being restrained by the disease due to the lack of cure and limited cosmetic resources (15). As in other stigmatized diseases, low self-esteem and internalized stigma can have devastating consequences on social interactions, vocational development, employment, and healthcare seeking (16).

### Health-Related Quality of Life

HRQL is a multi-dimensional concept that includes domains of physical, mental, emotional, and social functioning, and the social context in which people live (17). In chronic diseases, HRQL has become increasingly important in the assessment of disease severity, the evaluation of interventions, and the allocation of resources. A growing body of research indicates that CLE has a substantial negative impact on the physical, mental, and social health of people living with these conditions (12, 18–20). One of the instruments most commonly used to measure HRQL in CLE is the Skindex 29+3, a skin-specific validated scale that provides separate scores for three skin-related domains (symptoms, emotions, and functioning) and an additional lupus-specific domain to address a patient's worries about hair loss, outdoor activities, and photosensitive-related flares (18, 21). The impact of CLE on the HRQL has been reported to be worse or similar to that seen in patients with other skin diseases, such as acne and non-melanoma skin cancer, as well as in other chronic conditions such as cardiovascular disease and diabetes (18).

HRQL can be influenced by multiple factors, including CLE subtypes and disease characteristics, patient's demographics, social context, and healthcare system. Female sex, older age, low education, low socioeconomic status, smoking, associated SLE, generalized CLE, and higher skin disease activity, have been reported to impact negatively different domains of HRQL in CLE (12, 18–20, 22, 23). Increased disease activity has been associated with poorer quality of life in cross-sectional studies; however, a small longitudinal study among patients with DLE and SCLE pointed to a physician-patient dissociation of the disease assessment, supporting the multidimensional patient-driven nature of quality of life in chronic skin diseases (24). A more recent study used a CLE-specific tool derived from Skindex 29+3 to examine multiple factors potentially associated with the HRQL in a diverse university-based sample of CLE patients from the Southwest US (20). Pain, fatigue, disease activity, body image, and side effects of medications were significantly associated with worse quality of life, with body dissatisfaction having the highest negative impact. These results taken together suggest that treatment evaluation should include measures relevant to the patient, including body appearance.

### Depression and Psychiatric Disorders

Psychological health is one of the HRQL domains most negatively impacted in CLE. Patients with CLE have increased prevalence of major depressive disorder, generalized anxiety disorder, panic disorder, suicide risk, and agoraphobia (25). Approximately one-third of people with CLE report moderate to severe depressive symptoms (18, 26–28). Likewise, the risk of depression was found to be 2-fold higher people with CLE compared with the general population in a nationwide Danish study (27). However, mental health challenges are often underdiagnosed and remain untreated in CLE patients and the psychosocial burden of CLE is poorly understood, particularly among patients from minority groups (28, 29).

While the CLE subtype and morphological characteristics are deemed to be primary factors affecting patients' quality of life, recent research suggests that individual characteristics and social factors also play critical roles (20). A study on illness perception among patients with DLE emphasized that negative emotional reactions to illness are associated with worse quality of life, worse depression and higher activity and damage (30). Furthermore, in a predominantly Black population-based cohort of patients with CCLE, the risk of depression was lower in participants who were employed and insured. Non-depressed patients also reported higher social support, visited a primary care physician more frequently in the last year, and reported better physician-patient interactions (28). Perceptions of stigmatization have been significantly related to both psychological distress and degree of disability among patients with other skin diseases (31–33), and these factors are likely to play a substantial role in the pathogenesis of depression among individuals with CCLE. Despite the high prevalence of depression in patients with CLE, in general, and CCLE, in particular, there is currently sparse work exploring psychosocial pathways in high-risk populations with CLE.

### Social Determinants of Health and CLE

The World Health Organization defines social determinants of health (SDH) as the conditions in which people are born, grow, live, work, and age that affects a wide range of health and quality-of life-risks and outcomes. SDH are narrowly correlated to the immediate environment of an individual such as underprivileged social conditions of poverty, lower level of education, unemployment, insecure housing, unsafe home and neighborhood conditions, unsafe employment, childhood experiences (e.g., abuse), poor relationships, and social support (34). Not only do SDH shape individuals' options, choice, and behavior that impact their health, but these conditions also correlate with environmental and social threats that generate unhealthy stress responses. Among patients with chronic skin diseases, social stigma and reduced social connections have been significantly related to both psychological distress and disability (33). However, little is known about the impact of SDH in CLE. Moreover, as Black individuals are at higher risk for chronic disfiguring subtypes and are also more likely to be exposed to social stressors, it is imperative to examine the impact of SDH on the health of this population.

A recent report from the University of Texas Southwestern CLE Registry examined the cross-sectional association of income and quality of life in an ethnically diverse sample of patients with CLE, of whom nearly 80% had DLE and 51% had associated SLE (13). Racial disparities in annual income were evident, with White people representing nearly 60% of participants in the highest bracket (>50 K USD) and Black people representing nearly 70% of those in the lowest bracket (<10 K USD). While Cutaneous LE Disease Area and Severity Index (CLASI) activity scores did not differ significantly across income, CLASI damage scores and income were inversely associated. Moreover, lower annual income was significantly associated with worse quality of life, specifically in relation to symptoms and emotions, and within those in the lowest income bracket, women, patients younger than 40 years of age, smokers, and those with more active skin disease were more likely to have worse quality of life. These findings suggest that CLE conditions place a substantial financial burden on patients, potentially limiting job opportunities and having negative consequences on healthcare access and quality of care. Moreover, low-income individuals reportedly experienced more shame, anger, embarrassment and social isolation related to their skin disease, suggesting that individuals living under the poverty threshold are disproportionately more vulnerable to the psychological and social effects of these stigmatizing conditions.

### Burden on the Health Care System

A recent study using administrative data indicated that CLE poses a substantial toll on the healthcare system. The total direct medical cost associated with CLE in the US was ~$30 billion in 2014, and CLE patients with depression had significantly higher average annual total expenditure, compared to those without depression ($19,854 vs. 9,735) (26).

### Cardiovascular Disease

A large body of evidence indicates that SLE and related autoimmune diseases increase the risk of cardiovascular disease (CVD), primarily as a consequence of immune-driven atherosclerotic changes (35–38). Recent research suggests that patients with isolated CLE may also have an increased risk of CVD, although data from various CLE studies are less consistent than in SLE (39–41). An increased cardiovascular risk in CLE can be explained by the chronic inflammatory process that characterize CLE, as well as by the high prevalence of depression in this population, which is a well-known factor associated with atherosclerosis (25, 42). Moreover, traditional risk factors, such as smoking and alcohol intake are coping responses frequently adopted by patients with stigmatized conditions, such as CLE (43). Less known factors, not studied yet in CLE, are related to the chronic exposure to psychosocial stressors, such as social stigma and discrimination. The experience of psychosocial stressors across the life-course contributes to “weathering”, or accelerated declines in health due to cumulative burden on biological systems (44–47). Research suggests that chronic stressors elicit a cascade of biological responses that may be functional in the short term, but over time damage the systems that regulate the body's stress response (48–50). Epidemiological studies have shown that psychological stress may significantly contribute to the development and progression of atherosclerosis (51–53).

### Relationship of CLE and SLE

ACLE lesions often present as a cutaneous flare within the context of SLE, whereas up to 60% of SCLE patients may have associated systemic features or may transition to SLE (54). In contrast, CCLE conditions in general and DLE in particular are deemed to have a lower risk of associated SLE or disease progression. Still, available data vary widely depending on the demographics and settings of the study population, methods and timing used to ascertain cases, and case definitions. The prevalence DLE lesions in patients with a diagnosis of SLE ranges between 5 and 24% (6, 55, 56), and similar proportions (5–25%) of patients with isolated DLE may progress to SLE (6, 9, 54, 57). Several studies indicate that when systemic manifestations are present in patients with DLE, these tend to be mild and kidneys are less likely to be compromised (56–59). The time from DLE to SLE progression varies widely, ranging between months to over 30 years (9, 57). One study described that nearly 17% of patients with a diagnosis of DLE developed SLE within 3 years (6), and the highest rates of disease progression within 3 years of DLE diagnosis have been reported for children (26%) and women (20.7%) (6, 55). However, a recent retrospective study underlined a much shorter estimate, with a median interval of 453 days between DLE diagnosis and SLE progression in 34 adult DLE patients who developed SLE (60). The progression from DLE to SLE has been linked to several clinical risk factors, including the presence of generalized DLE lesions, articular symptoms (arthritis or arthralgias), periungual telangiectasias and nailfold abnormalities, autoantibodies, leukopenia and anemia (61, 62). The pathogenic mechanisms for SLE progression are largely unknown. A recent cross-sectional study in a predominantly Black population underlined that the B-cell compartment in some patients with isolated CCLE resembles SLE and is clinically associated with enhanced serological activity and more extensive skin disease, suggesting that SLE-like B-cell changes may help identify CCLE patients at risk for subsequent development of SLE (63). In contrast, another study found a B cell gene signature in the skin of DLE patients, which was more prominent in patients with a lower rate of systemic disease. These findings taken together suggest that B cell phenotypes in the blood and the skin may play specific roles with differential effect in cutaneous lupus and systemic disease activity.

## Unmet Needs and Research Opportunities

Qualitative studies among CLE patients revealed important unmet needs related to CLE treatment and care, including insufficient patient education to better cope with the disease and lack of treatments to improve damaged skin (14). Furthermore, Black patients tend to report low satisfaction with dermatologists' knowledge of their skin and hair, as well as lack of culturally sensitive interaction style. Since Black people are more susceptible to DLE than White people and are more likely to develop lesions on the scalp with more severe damage and dyspigmentation, a knowledgeable and culturally competent approach is necessary to better serve these patients. Cosmetic care is another unmet need perceived by patients. Cosmetic procedures are largely avoided by practitioners because the potential side effects that may occur in autoimmune and photosensitive conditions. Moreover, these procedures are expensive and patients with CCLE are often left with permanent skin damage (64).

Despite the heterogeneous spectrum of CLE conditions, as well as the variable disease severity and risk of systemic manifestations across these multiple conditions, most quality-of-life studies tend to approach CLE as a group, with limited data on the potential differences by CLE subtypes. Furthermore, the susceptibility to CLE subtypes and the disease severity differs by individual demographics, with Black patients having higher risks of chronic subtypes, more conspicuous hypopigmentation, and worse skin damage (8, 13, 19). Thus, studies with larger sample size and representation of minority groups are needed to better describe health disparities across CLE subtypes and understand the needs of patients from vulnerable groups.

The study of social determinants has been lacking and is fundamental in CLE, where CCLE, the most prevalent subtype, clearly disproportionately strikes Black minorities. Research addressing social determinants of health is imperative to understand the pathways associated with poor outcomes and inform clinicians, public health agents and general public on interventions and programs that can help to mitigate the negative impact of these conditions in the most vulnerable subpopulations.

## Author Contributions

CD and SL contributed to the manuscript conception and literature review. All authors were involved in drafting the article and/or critically revising it for important intellectual content, and approved the final version to be published.

## Funding

SL and CD are supported by the NIH (R01AR065493-01; R01MD010455-01; R01AR070898-01) and the CDC (U01DP005119). This publication was supported by the Centers for Disease Control and Prevention of the U.S. Department of Health and Human Services (HHS) under Grant numbers U01 DP19003, U01 DP005119 and by cooperative agreement CDC-RFA-DP08-806 as part of a financial assistance award totaling $6,406,636 with 100% funded by CDC/HHS.

## Author Disclaimer

The findings and conclusions in this report are those of the authors and do not necessarily represent the official position of the CDC.

## Conflict of Interest

The authors declare that the research was conducted in the absence of any commercial or financial relationships that could be construed as a potential conflict of interest.

## Publisher's Note

All claims expressed in this article are solely those of the authors and do not necessarily represent those of their affiliated organizations, or those of the publisher, the editors and the reviewers. Any product that may be evaluated in this article, or claim that may be made by its manufacturer, is not guaranteed or endorsed by the publisher.
